# Use of Symptomatic Drug Treatment for Fatigue in Multiple Sclerosis and Patterns of Work Loss

**DOI:** 10.1002/acn3.70259

**Published:** 2025-11-20

**Authors:** Simon Englund, Johan Reutfors, Thomas Frisell, Fredrik Piehl

**Affiliations:** ^1^ Department of Clinical Neuroscience Karolinska Institutet Stockholm Sweden; ^2^ Clinical Epidemiology Division, Department of Medicine Karolinska Institutet Stockholm Sweden

**Keywords:** amantadine, central stimulants, fatigue, modafinil, multiple sclerosis, work loss

## Abstract

**Objective:**

To describe the use of central stimulants and amantadine for fatigue in MS and evaluate a potential association with reduced work loss in people with MS.

**Methods:**

We conducted a nationwide, matched, register‐based cohort study in Sweden (2006 to 2023) using national registers with prospective data collection. We included individuals with MS, identified via the Swedish MS register or ≥ 3 MS diagnoses in the National Patient Register, who initiated treatment with modafinil, amantadine, or central stimulants for ADHD (ADHD‐Drugs), along with untreated individuals matched on modafinil start date, age, sex, time since MS diagnosis, and prior‐year work loss. The main outcome was monthly work loss, defined as the sum of net days on sick leave, disability pension, and activity compensation.

**Results:**

We identified 2162 new modafinil users, 462 amantadine, 424 ADHD drugs, and 9762 untreated. All cohorts showed increasing work loss before index, followed by no change in work loss over the subsequent 24 months. Modafinil users had a significantly greater attenuation, but with low effect size, of the increasing trajectory of average monthly work loss than untreated (−0.17 days; 95% CI: −0.22, −0.12), with no significant differences between modafinil and other treated groups. Modafinil was the most prescribed treatment, with 1‐year prevalence of 8.5% in 2006 and 7% in 2023.

**Interpretation:**

A potential minor treatment benefit is suggested by the statistically significant, small attenuation of worsening work loss following fatigue treatment initiation compared with untreated people with MS. No differences in treatment benefit were observed across fatigue treatments.

## 1 Introduction

Fatigue is a prevalent and debilitating symptom affecting up to two‐thirds of people with multiple sclerosis (PwMS) [1, 2], with a significant negative impact on quality of life and daily functioning [2]. Importantly, fatigue is one of the strongest predictors of work loss in MS [3, 4, 5], since PwMS suffering from fatigue experience difficulties maintaining capacity throughout a full workday, often resulting in sick leave and, in some cases, a disability pension [4, 6, 7].

Both pharmacological and non‐pharmacological strategies are used to manage fatigue in MS [8], although no drug is specifically approved for MS fatigue. Drugs such as amantadine, modafinil, and amphetamine‐like CNS stimulants are nevertheless used off‐label to a largely unknown extent [9].

Some PwMS reportedly experience fatigue relief from these drugs [8], but high‐quality clinical evidence is scarce [8, 10, 11]. Randomized controlled studies have yielded conflicting results, often due to small sample sizes and short follow‐up [8, 10, 12, 13]. Moreover, the subjective nature of fatigue assessments, combined with noticeable off‐target effects, may lead to placebo effects [14, 15, 16, 17]. Importantly, central stimulants may also have adverse effects, such as on the cardiovascular system [18, 19]. In summary, although recent evidence suggests that the efficacy of symptomatic drug treatment for MS fatigue is minimal or modest [20, 21], these drugs are still used in clinical practice, likely reflecting an important unmet medical need.

The objectives of this study were to quantify the use of central stimulants and amantadine as symptomatic treatments for MS fatigue (“fatigue treatments”) using nationwide prescription data, and to evaluate a potential association with reduced work loss in PwMS. Specifically, the four following key research questions were formulated: (i) What is the annual prescription rate of commonly used treatments for fatigue in Sweden? (ii) How frequently do PwMS refill prescriptions for fatigue treatments within two years of initiation? (iii) What are the characteristics of new users and nonusers of fatigue treatments? (iv) What is the impact of initiating fatigue treatments on monthly work loss rates, both across different treatments and in comparison with untreated PwMS?

## 2 Methods

This nationwide, matched, register‐based cohort study included PwMS identified from the Swedish National Patient Register (NPR) [22] and the Swedish MS Register (SMSreg) [23]. Data were linked to other Swedish National Healthcare and Census Registers using each individual's unique Swedish identification number [24]. The linked registers included the Prescribed Drug Register (PDR) [25], the Swedish Social Insurance Agency's database, Micro Data for Analysis of the Social Insurance (MiDAS), the Population Register [26], and the longitudinal database for insurance and labor market studies (LISA) [27].

The study was approved by the Swedish Ethical Review Authority (Dnr 2021‐02384, with amendment 2023‐07906‐02).

### 2.1 Data Sources

SMSreg is a non‐compulsory web‐based registry collecting data on MS patients in Sweden since 2001, with approximately 80% coverage of PwMS in 2015 [23]. The NPR contains all registered International Classification of Diseases (ICD) codes from hospital discharges since 1987, and non‐primary outpatient visits since 2001 [22]. The PDR records all dispensations of prescribed drugs in Sweden since 2005, including Anatomical Therapeutic Chemical (ATC) codes [25].

### 2.2 Study Population

**FIGURE 1:**
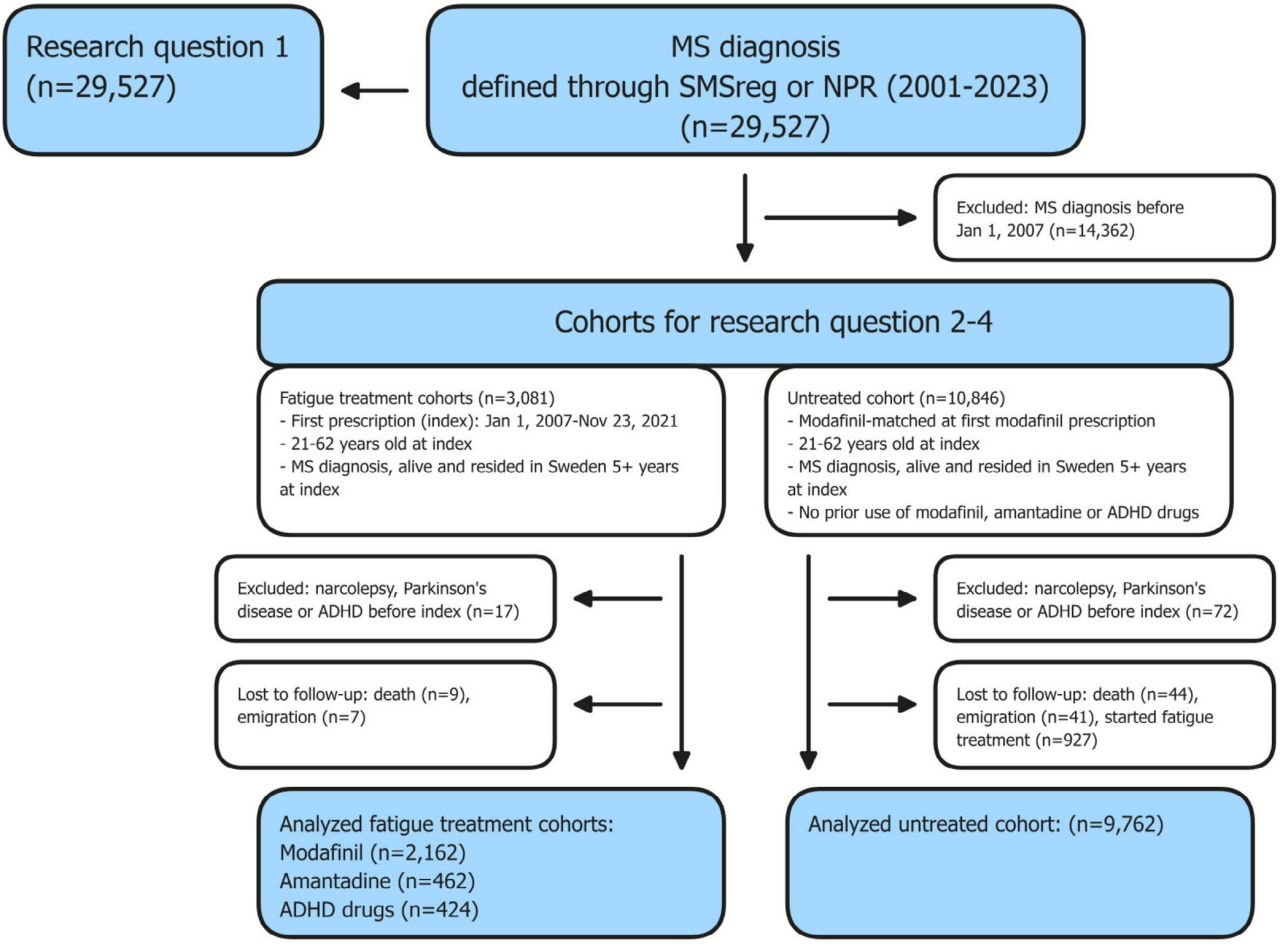
Study flowchart. Flowchart illustrating the inclusion, follow‐up, and analysis for the following research questions: (i) What is the annual prescription rate of commonly used treatments for fatigue in Sweden? (ii) How frequently do PwMS refill prescriptions for fatigue treatments within two years of initiation? (iii) What are the characteristics of new users and nonusers of fatigue treatments? (iv) What is the impact of initiating fatigue treatments on monthly work loss rates, both across different treatments and in comparison with untreated PwMS? ADHD, attention deficit hyperactivity disorder; MS, multiple sclerosis; NPR, the Swedish National Patient Register; SMSreg, Swedish MS registry.

Figure 1 presents the study flowchart, with the different study cohorts. A total of 29,257 PwMS were included, identified through SMSreg or by ≥ 3 separate MS diagnoses (ICD‐10 G35.9) in the NPR [28]. If available, the SMSreg diagnosis date was used; otherwise, the first occurrence in NPR was used. To estimate prescription rates over time, the annual population included individuals residing in Sweden from January 1 to December 31 of each study year (2006–2023), with an MS diagnosis recorded during or before that year. For the other research questions, we identified 14,464 PwMS diagnosed on or after January 1, 2007, ensuring > 1 year of follow‐up since the launch of PDR in July 2005.

### 2.3 Definition of Fatigue Treatment Cohorts and the Untreated Cohort

Fatigue treatments were identified in the PDR using filled prescriptions for modafinil (N06BA07), amantadine (N04BB01), and attention deficit hyperactivity disorder (ADHD) drugs of the central stimulant type including amfetamine (N06BA01), dexamfetamine (N06BA02), methylphenidate (N06BA04), and lisdexamfetamine (N06BA12). Prescriptions issued by psychiatrists were excluded. Individuals could contribute to multiple treatment cohorts during different study periods.

For research questions 2–4, fatigue treatment cohorts were defined by the first filled prescription of each treatment, referred to as the index date, between January 1, 2007, and November 23, 2021. To ensure new use, individuals were required to have no previous dispensed prescription for the treatment since the launch of the PDR. Risk‐set sampling was used to match first‐time modafinil users to up to five untreated pwMS untreated at the index date. Matching variables included sex, age (±1 year), years since MS diagnosis (±1 year), and prior‐year work loss (yes/no). Matched controls had no prior use of modafinil, amantadine, or ADHD drugs and could serve as controls for multiple modafinil users. They were also required to remain untreated for fatigue during the 24‐month follow‐up period post‐index. Additional inclusion criteria included age 21 to 62 years and residency in Sweden for at least 5 years before the index date. PwMS with a diagnosis of ADHD (ICD‐10 F90), narcolepsy (G47.4), or Parkinson's disease (G20.9) before the index date were excluded. Observations were censored upon death, emigration from Sweden, or the end of the follow‐up period (November 23, 2023), whichever occurred first. An ever‐treated approach was applied to the fatigue treatment cohorts, whereby all individuals were followed from the start of treatment up to 24 months post‐initiation, regardless of whether they later discontinued or switched treatments.

### 2.4 Outcome Definition

The primary outcome for research question 4 was work loss, as derived from MiDAS' records, as a unified measure combining days of sick leave, disability pension, and “activity compensation.” Sick leave substitutes short to medium‐term income loss, while disability pension and activity compensation provide long‐term support for sustained work limitations. Combining these benefits enhances consistency and resilience to institutional changes that may shift individuals between these systems. Benefits can be granted at 25%, 50%, 75%, or 100% of full‐time employment, and were converted to net days.

In Sweden, all employees, including those on parental leave or receiving unemployment benefits, are entitled to sick leave benefits. Employers typically cover Day 2 to Day 14, with the Swedish Social Insurance Agency covering longer absences. To ensure comparability across employment statuses, only sick leave episodes exceeding 14 days were included in the analysis.

### 2.5 Covariates

Covariates included in the multiple imputation and propensity score models were: age, sex, region of residence, country of birth, education, days of work loss, employment status, history of hospitalized days, and prior year use of treatments including antidepressants, anxiolytics, sleeping aids, pain treatment, and antidiabetics. Additionally, we included diagnoses of depression, anxiety disorder, other psychiatric comorbidities, sleep disorder, major acute cardiovascular event, arrhythmia, malignancy, and hospitalized infections, as well as MS type, disease duration, history of relapse, and use of disease‐modifying therapy (DMT). See Table S1 for detailed variable definitions.

### 2.6 Statistical Analysis

Generalized estimating equations (GEE) were conducted to estimate the impact of initiating fatigue treatments on subsequent work loss. Using data from 12 months before to 24 months after the index date, we compared: (1) change in monthly work loss in each cohort before and after treatment initiation, and (2) differences in the rate of changes between cohorts. Modafinil was the reference for comparisons across the fatigue treatment cohorts, while the untreated cohort served as a reference for comparisons with the fatigue treatment cohorts. Because untreated individuals were matched specifically to modafinil initiation, direct comparisons with amantadine and ADHD drugs are not straightforward. Robust standard errors (Huber–White) were calculated to account for weighting and the possibility of individuals contributing to multiple cohorts.

Missing covariate values were addressed with multiple imputations by chained equations with 20 imputations and 10 burn‐in iterations. Continuous variables were imputed using predictive mean matching, and categorical variables with multinomial logistic regression. Estimates were combined using Rubin's rules, providing 95% confidence intervals (CIs) based on the normal approximation. The degree of missing data is detailed in Table S2.

Stabilized inverse probability of treatment weighting (IPTW) was applied to adjust for cohort imbalances [29]. Weights were calculated by multiplying the overall sample proportion of individuals in each cohort by the inverse probability cohort membership, estimated using multinomial logistic regression, and truncated at the 99th percentile. Continuous variables were modeled as second‐degree (age, days of work loss in the last year, and days hospitalized in the last year) and third‐degree (years since MS diagnosis) polynomials. Other variables were treated as categorical, and interactions between age and sex were included. Standardized mean differences (SMDs) were calculated to assess balance before and after weighting. Region of residence, days of work loss, years since MS diagnosis, relapse history, and use of DMT; interferons, showed SMDs outside the range of −0.1 to 0.1 (Figure S1).

All statistical analyses were performed using SAS Enterprise Guide 8.3.

### 2.7 Sensitivity Analyses

Two prespecified sensitivity analyses were conducted to assess the risk of confounding in the main model. First, we added MS severity‐related covariates excluded from the main IPTW model due to high missingness; physical disability scored by Expanded Disability Status Scale (EDSS) [30], cognitive processing speed with Symbol Digit Modalities Test (SDMT) [31], and self‐reported physical and psychological impact assessed with the MS Impact Scale 29 (MSIS‐29) [32], all modeled with second degree polynomials. These covariates were included in both the IPTW model and the multiple imputation procedure (Figure S2). Second, we applied a doubly robust GEE model involving adjustments at both the weighting and the modeling stage. Region of residence, days of work loss, years since MS diagnosis, relapse, and DMT were included as covariates in addition to being accounted for in the IPTW weighting. At reviewer request, an analysis of weighted mean monthly work loss in each cohort from 12 months before to 24 months after the index date was stratified into pre‐2020 and 2020–2023 to account for workplace disruptions during the pandemic.

## 3 Results

### 3.1 Annual Fatigue Treatment Prescription Rate

**FIGURE 2:**
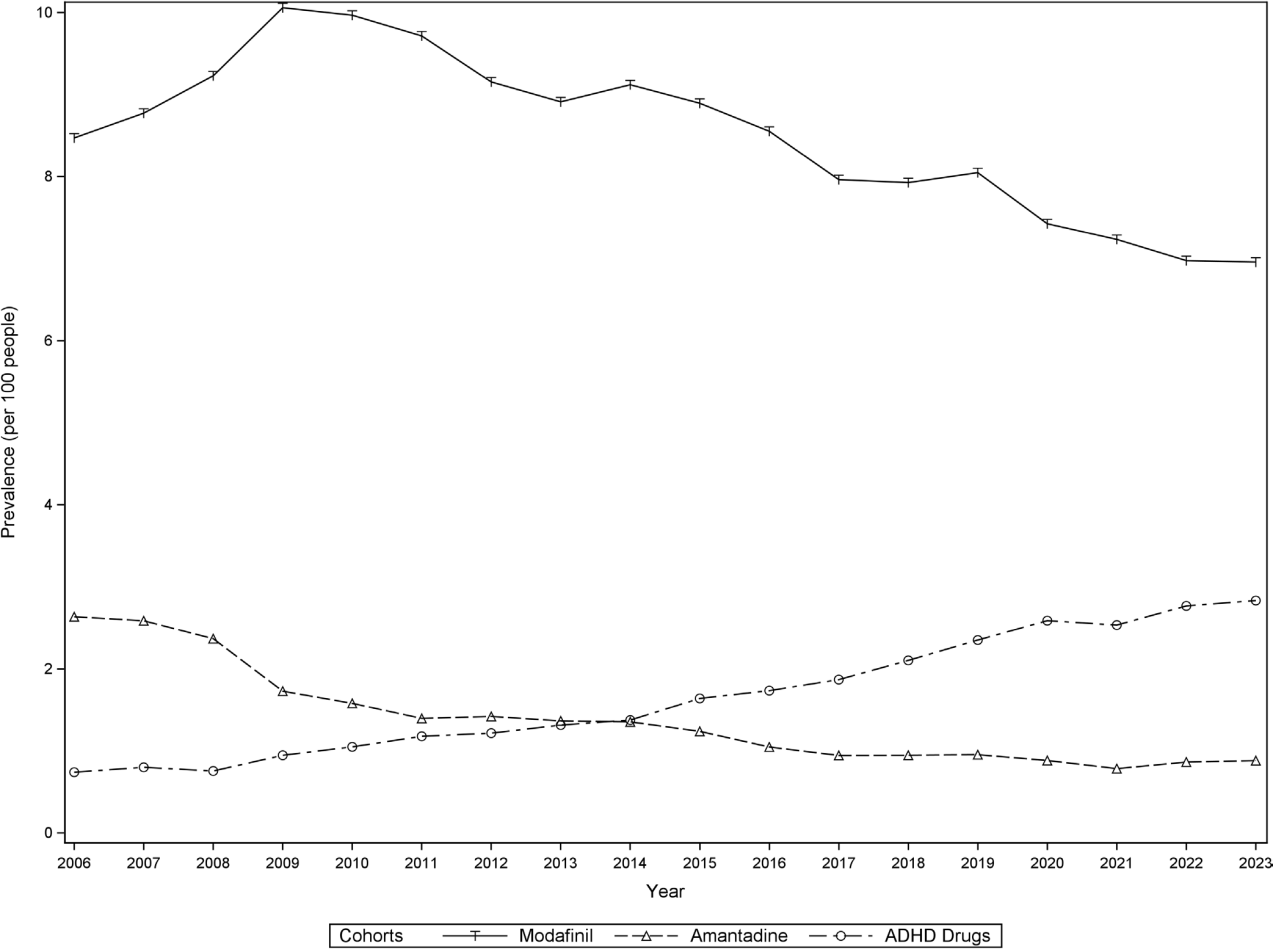
Annual prescription rates of modafinil, amantadine, and ADHD drug use among people with MS from 2006 to 2023 in Sweden. Period prevalence was calculated as the number of PwMS who filled at least one prescription per year, divided by the at‐risk MS population. Prescriptions issued by psychiatrists were excluded. ADHD, attention deficit hyperactivity disorder.

The annual period prevalence of modafinil, amantadine, and ADHD drug use among PwMS from 2006 to 2023 is illustrated in Figure 2. In 2006, the 1‐year prevalence of modafinil use was 8.5%, increasing to 10% in 2009, and then declining to 7% in 2023. The use of amantadine also declined, from 2.6% in 2006 to 0.9% in 2023, while ADHD drug use increased from 0.7% in 2006 to 2.8% in 2023. Overall, the proportion of PwMS prescribed any fatigue treatment remained relatively stable, decreasing from 11.3% in 2006 to 10.2% in 2023.

### 3.2 Baseline Characteristics at Index Date and Rate of Re‐Filled Prescriptions

For subsequent analyses, we identified 2162 new modafinil users, 462 new amantadine users, 424 new ADHD drug users, and 9762 matched untreated individuals. Overall, 72.0% were female, 86.7% had RRMS, with a mean age of 41.3 years (standard deviation [SD] 10.0) and 2.8 years (SD 2.6) since MS diagnosis at the index date. Characteristics were similar across cohorts in terms of age, MS type, and sex. However, the untreated cohort had the highest education and lowest rates of depression, anxiety, and pain treatment, whereas work loss days and psychiatric disorders, with related prescriptions, were more pronounced in treated cohorts, especially the ADHD drug group (Table 1). Most new modafinil users (95.1%) had no prior fatigue treatment prescriptions, compared to 48.1% of new amantadine users and 11.1% of new ADHD drug users. The latter group also had the longest MS disease duration (4.4 years) and the lowest proportion of relapses in the past year (7.7%).

**TABLE 1:** Characteristics at index date for fatigue treatment cohorts and the untreated modafinil‐matched MS cohort.

	Cohorts			
	Untreated	Modafinil	Amantadine	ADHD drugs^a^
No. of participants	9762	2162	462	424
Included in SMSreg, *n* (%)	8801 (90.2)	2089 (96.6)	448 (97.0)	417 (98.3)
Index year^b^, median (IQR)	2015 (2012–2018)	2015 (2011–2018)	2015 (2012–2018)	2018 (2015–2020)
Age, year, mean (SD)	41.4 (10.0)	41.3 (10.0)	40.9 (9.6)	40.6 (9.3)
Sex, female, *n* (%)	7025 (72.0)	1559 (72.1)	336 (72.7)	305 (71.9)
Swedish‐born, *n* (%)	8446 (86.5)	1929 (89.2)	424 (91.8)	376 (88.7)
Education > 12 years, *n* (%)	4177 (43.0)	850 (39.4)	170 (37.0)	179 (42.2)
Unemployed, *n* (%)	565 (5.8)	148 (6.8)	27 (5.9)	30 (7.1)
Days of work loss^c^ last year, mean (SD)	109.4 (137.4)	118.2 (134.0)	135.2 (140.8)	158.3 (144.7)
Days hospitalized last 5 years, mean (SD)	9.4 (35.8)	7.5 (14.8)	7.9 (22.3)	9.8 (20.2)
Depression^d^, *n* (%)	662 (6.8)	189 (8.7)	48 (10.4)	51 (12.0)
Anxiety disorder^d^, *n* (%)	943 (9.7)	212 (9.8)	42 (9.1)	56 (13.2)
Other psychiatric comorbidities^d^, ^e^, *n* (%)	712 (7.3)	155 (7.2)	29 (6.3)	35 (8.3)
Sleep disorders^d^, *n* (%)	146 (1.5)	36 (1.7)	9 (1.9)	14 (3.3)
MACE^d^, *n* (%)	169 (1.7)	40 (1.9)	6 (1.3)	2 (0.5)
Arrhythmia^d^, *n* (%)	127 (1.3)	22 (1.0)	5 (1.1)	7 (1.7)
Any invasive cancer^d^, *n* (%)	123 (1.3)	32 (1.5)	6 (1.3)	3 (0.7)
Any hospitalized infection^d^, *n* (%)	469 (4.8)	106 (4.9)	25 (5.4)	28 (6.6)
Antidepressant use^f^, *n* (%)	1767 (18.1)	565 (26.1)	140 (30.3)	156 (36.8)
Anxiolytics treatment use^f^, *n* (%)	466 (4.8)	126 (5.8)	41 (8.9)	30 (7.1)
Sleeping aids treatment use^f^, *n* (%)	1638 (16.8)	483 (22.3)	106 (22.9)	101 (23.8)
Pain treatment use^f^, *n* (%)	4139 (42.4)	1191 (55.1)	259 (56.1)	236 (55.7)
Antidiabetic use^f^, *n* (%)	335 (3.4)	65 (3.0)	14 (3.0)	12 (2.8)
Fatigue treatment order				
First	—	2056 (95.1%)	222 (48.1%)	47 (11.1%)
Second	—	106 (4.9%)	224 (48.5%)	281 (66.3%)
Third	—	0 (0.0%)	16 (3.5%)	96 (22.6%)
MS type, *n* (%)				
RRMS	7640 (87.6%)	1751 (84.4%)	372 (84.0%)	342 (82.4%)
SPMS	455 (5.2%)	153 (7.4%)	36 (8.1%)	44 (10.6%)
PPMS	631 (7.2%)	171 (8.2%)	35 (7.9%)	29 (7.0%)
Years since MS diagnosis, mean (SD)	2.7 (2.6)	2.6 (2.6)	3.0 (2.7)	4.4 (3.1)
Any relapse last year, *n* (%)	1144 (15.1)	314 (17.3)	54 (14.0)	31 (7.7)
DMT, *n* (%)				
Dimethyl fumarate	696 (9.2%)	180 (9.9%)	29 (7.5%)	37 (9.2%)
Fingolimod	334 (4.4%)	91 (5.0%)	15 (3.9%)	32 (8.0%)
Glatiramer acetate	388 (5.1%)	111 (6.1%)	28 (7.2%)	12 (3.0%)
HSCT	91 (1.2%)	6 (0.3%)	3 (0.8%)	9 (2.2%)
Interferons^g^	1534 (20.3%)	343 (18.8%)	76 (19.6%)	26 (6.5%)
Natalizumab	1039 (13.7%)	271 (14.9%)	69 (17.8%)	64 (16.0%)
Rituximab	1843 (24.3%)	479 (26.3%)	99 (25.6%)	159 (39.7%)
Teriflunomide	123 (1.6%)	28 (1.5%)	5 (1.3%)	4 (1.0%)
Other^h^	106 (1.4%)	31 (1.7%)	5 (1.3%)	15 (3.7%)
No DMT	1416 (18.7%)	280 (15.4%)	58 (15.0%)	43 (10.7%)

Abbreviations: ADHD, attention deficit hyperactivity disorder; DMT, disease modifying therapy; HSCT, hematopoietic stem cell transplantation; IQR, interquartile range; MS, multiple sclerosis; PPMS, primary progressive MS; RRMS, relapsing remitting MS; SD, standard deviation; SMSreg, Swedish MS registry; SPMS, secondary progressive MS.

^a^ Central stimulants for ADHD amfetamine, dexamfetamine, methylphenidate, and lisdexamfetamine.

^b^ Index year corresponds to the first filled prescription for each treatment cohort, and for the untreated cohort, it is the year of the matched modafinil prescription.

^c^ Number of net days with sick leave, disability pension or activity compensation.

^d^ Any diagnosis in the last 5 years.

^e^ All mental and behavioral disorders except depression and anxiety disorders.

^f^ Any filled prescription during the last year.

^g^ Interferon beta‐1a and interferon beta‐1‐b.

^h^ Alemtuzumab, cladribine, ocrelizumab, ofatumumab, and siponimod.

Among new users of modafinil and ADHD drugs, 27.8% and 15.1%, respectively, did not refill a prescription within the first 2 years (i.e., they filled only a single prescription), while the corresponding proportion for amantadine was 49.6%.

### 3.3 The Impact on Work Loss

**FIGURE 3:**
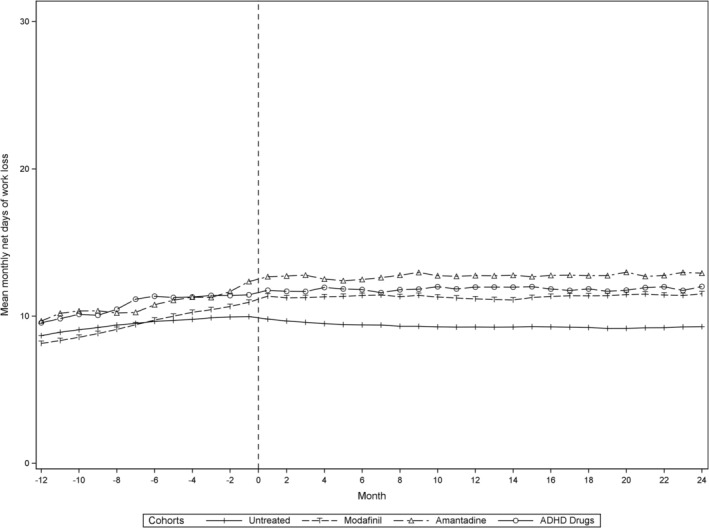
Weighted mean monthly net days of work loss from 12 months before to 24 months after the index date. Weights from stabilized inverse probability of treatment weighting were used to account for baseline imbalances between cohorts at the index date. ADHD, attention deficit hyperactivity disorder.

Figure 3 illustrates the weighted mean monthly work loss from 12 months before to 24 months after the index date, while Table 2 details GEE‐based estimates of the weighted mean monthly work loss before index (intercept), and change over time. All fatigue treatment cohorts showed similar trajectories, with a linear increase in average monthly work loss of 0.18 to 0.29 days during the year before index, followed by no change over the subsequent 2 years. This pattern was also observed, albeit less pronounced, in the untreated reference cohort. The reduction in the increasing trajectory of average monthly work loss was significantly greater in the modafinil cohort compared to the untreated cohort, with a difference of −0.17 days (95% CI: −0.22 to −0.12). Corresponding estimates for the amantadine and ADHD drug cohorts compared to the untreated cohort were −0.12 days (95% CI: −0.26 to 0.01) and −0.08 days (95% CI: −0.21 to 0.06), respectively. Across treated cohorts, no statistically significant differences were found in changes in the trajectory of work loss between the pre‐ and post‐index periods for the amantadine and ADHD drug cohorts relative to the modafinil cohort.

**TABLE 2:** Weighted mean monthly work loss pre‐index and changes in work loss rates during pre‐index and from pre‐ to post‐index, within and between cohorts.

	Mean monthly work loss, (95% CI)	Change in monthly work loss rates, Mean (95% CI)	
	Pre‐index	Pre‐index	Pre‐ to post‐index
Cohort			
Untreated	9.76 (9.51; 10.01)	0.07 (0.05; 0.09)	−0.10 (−0.12; −0.08)
Modafinil	11.15 (10.59; 11.70)	0.29 (0.24; 0.33)	−0.27 (−0.32; −0.22)
Amantadine	12.33 (10.95; 13.71)	0.26 (0.15; 0.36)	−0.23 (−0.36; −0.10)
ADHD drugs^a^	11.78 (10.32; 13.23)	0.18 (0.08; 0.29)	−0.18 (−0.31; −0.05)
Comparison between cohorts			
Modafinil vs. Untreated	1.38 (0.78; 1.99)	0.21 (0.17; 0.26)	−0.17 (−0.22; −0.12)
Amantadine vs. Untreated	2.56 (1.16; 3.96)	0.19 (0.08; 0.29)	−0.12 (−0.26; 0.01)
ADHD drugs^a^ vs. Untreated	2.01 (0.53; 3.49)	0.11 (0.01; 0.22)	−0.08 (−0.21; 0.06)
Amantadine vs. Modafinil	1.18 (−0.31; 2.67)	−0.03 (−0.14; 0.09)	0.04 (−0.10; 0.18)
ADHD drugs^a^ vs. Modafinil	0.63 (−0.93; 2.19)	−0.10 (−0.22; 0.01)	0.09 (−0.05; 0.24)

*Note*: A positive number corresponds to a net loss of work capacity. Estimates are derived from a generalized estimating equations model, incorporating weights from stabilized inverse probability of treatment weighting to account for imbalances between cohorts at index date. Weighting was applied for age, sex, country of birth, region of residence, education, employment status, work loss, hospitalized days, depression, anxiety, other psychiatric comorbidities, sleep disorders, MACE, arrhythmia, invasive cancer, hospitalized infection, antidepressants, anxiolytics, sleeping aids, pain treatments, antidiabetics, DMT, MS type, years since MS diagnosis, any relapse last year. The index date corresponds to the first filled prescription (new use) for each treatment cohort, and for the untreated cohort, it is the date of the matched modafinil prescription.

Abbreviations: ADHD, attention deficit hyperactivity disorder; CI, confidence interval.

^a^ Central stimulants for ADHD are amfetamine, dexamfetamine, methylphenidate, and lisdexamfetamine.

### 3.4 Sensitivity Analyses

Adjusting for measures of MS disease severity (EDSS, SDMT, and MSIS‐29) yielded results consistent with the main analysis for comparisons among the fatigue treatment cohorts (Table S3). For the comparison between the fatigue treatment cohorts and the untreated cohort, the additional adjustments attenuated the estimated pre‐post‐index changes but did not alter the interpretation of the results (Table S3). In the second sensitivity analysis, region of residence, years since MS diagnosis, recent relapses, and DMT were added as time‐stable covariates to the GEE model. As these covariates were time‐stable, only the intercepts across cohorts were affected (Table S4). Analyses stratified by index date (pre‐2020 and 2020–2023) revealed similar trajectories, except that the trajectories for amantadine and ADHD drugs were less stable due to the low number of treatment starts during 2020–2023, making interpretation of these results difficult (Figure S3). Overall, average monthly work loss was somewhat lower in 2020–2023 compared with the pre‐2020 period.

## 4 Discussion

In this nationwide, matched, register‐based cohort study on the use of symptomatic drug treatments for fatigue in PwMS in Sweden, modafinil was most commonly used, although ADHD drug prescription rates increased over time. All fatigue treatment cohorts displayed similar trajectories of average monthly work loss, shifting from an increasing trend before treatment initiation to no change after treatment initiation. A smaller change in trajectory was evident also in the untreated cohort, but the attenuation was significantly greater in the modafinil group (−0.17 days per month), corresponding to approximately 2 fewer days of work loss per year. The attenuations observed in the amantadine and ADHD drug groups, compared with the untreated group, were marginally smaller and not statistically significant. Comparisons of amantadine and ADHD drugs versus the untreated cohort should be interpreted cautiously, as the untreated group was matched specifically to modafinil initiation, making these comparisons not directly equivalent.

Modafinil was introduced as a narcolepsy treatment in Sweden in 2001 and soon became used off‐label for MS fatigue [33]. Amantadine is an older drug initially developed for influenza A, later being approved also for Parkinson's disease [34]. Prescribing practices in the United Kingdom indicate amantadine as a first‐line treatment for MS fatigue [8, 34]. However, our findings show that modafinil was the preferred first‐line treatment in Sweden, ADHD drug prescriptions increased over time and amantadine was least used. Notably, ADHD drug prescriptions by neurologists required an individual license from the Swedish Medical Products Agency before 2018 (similar to amphetamine throughout the whole study period), likely explaining their increase over time [35]. It cannot be excluded that some prescriptions may have been made for PwMS with suspected or confirmed ADHD without a registered diagnosis in the NPR, possibly due to incomplete capture of psychiatric diagnoses in the outpatient component of the NPR until around 2013. Additionally, some ADHD patients may have received ADHD drugs before a formal diagnosis. However, since PwMS who had a prior ADHD diagnosis or prescriptions issued by psychiatrists were excluded, the degree of potential bias can be expected to be limited. Finally, the yearly prevalence of modafinil increased between 2006 and 2009 but declined thereafter, likely following the European Medicines Agency's 2010 restriction of its use to narcolepsy, with or without cataplexy [36]. Modafinil had previously been prescribed for conditions associated with excessive sleepiness, such as obstructive sleep apnea and shift work sleep disorder. The decision was based on clinical trial data indicating a favorable benefit–risk profile only for adults with narcolepsy, with or without cataplexy [36]. This shift may also reflect a decline in off‐label use of modafinil for psychiatric conditions, following initial interest in the early 2000s [37].

There were certain differences between the treated cohorts and the matched controls. Firstly, treated individuals had higher rates of depression, anxiety, sleep difficulties, and pain, indicating a greater burden of MS‐related symptoms, psychiatric comorbidities, and polypharmacy. These findings align with our prior study [38], where PwMS with the highest self‐reported fatigue scores also had more comorbidities. Considering the established link between depression and fatigue [39], this is in line with the treated cohorts being enriched for people with greater fatigue.

Our finding of an inflection point for the work loss trajectory coinciding in time with modafinil initiation, and to a lesser extent with amantadine and ADHD drug initiation, suggests that pharmacological intervention may help some PwMS manage work responsibilities. However, due to study design, no definitive causal conclusions can be drawn. Key concerns include confounding by indication and regression to the mean due to the matching process. The risk of confounding by indication was reduced by comparing new users of fatigue treatments; however, comparisons between fatigue treatment users and untreated individuals carry a higher risk of confounding, as untreated individuals generally differ more from those who are treated. Causal interpretation can be strengthened in longitudinal observational studies when outcome levels (intercepts) and trajectories are parallel pre‐index. If the trajectories then diverge post‐index, this supports a potential causal effect. When we compared fatigue treatments to the untreated cohort, the pre‐index level of monthly work loss was significantly different, and the trajectories were not parallel. Although the trajectories diverged after index, the greater attenuation of monthly work loss in the modafinil group should be interpreted with caution due to these pre‐index differences. Regression to the mean arises in longitudinal analyses, and is particularly pronounced with only one pre‐ and one post‐intervention measurement, where extreme baseline values are likely to be followed by values closer to the average. In our study, assessing multiple measures over time both before and after treatment initiation mitigates this phenomenon. Nevertheless, the observed changes in work loss for the untreated group may reflect regression to the mean, particularly as matching was performed on prior work loss, potentially representing extreme values. Matching on work loss regardless of cause may also have biased comparisons.

The greater, yet minor, reduction in work loss trajectory associated with the start of modafinil may reflect a combined effect of the drug and non‐pharmacological interventions, such as fatigue management or physical training, which are first‐line options for MS fatigue [9]. Different types of fatigue (physical, cognitive, and psychological) may contribute differently to work functioning in PwMS, depending on the nature and demands of one's occupation. Some occupations allow for adjustments such as remote work or flexible schedules, whereas others offer limited opportunities for such adaptations, which may in turn influence the observed outcome [3]. Unfortunately, data on these non‐pharmacological interventions or on workplace accommodations were not available for this study. One should also consider that work loss typically increases at MS diagnosis, then stabilizes [4, 6].

To our knowledge, this is the first population‐based study using an objective (non‐self‐reported) outcome measure to compare fatigue treatments in PwMS. Partly consistent with previous, inconsistent evidence on short‐term fatigue treatment effects [8], we found no clear differences in work loss changes across treatment groups. Early double‐blind, placebo‐controlled crossover trials suggested modest efficacy of amantadine [15, 40, 41], but were limited by small sample sizes and short follow‐up [8]. Several single‐arm trials indicated a possible effect of modafinil [42, 43, 44], yet the absence of comparators renders the level of evidence low. In a double‐blind crossover trial, Rammohan et al. [45] found a benefit of 200 mg/day modafinil but not 400 mg/day, while other well‐designed trials provided little support for amantadine or modafinil over placebo [14, 46, 47]. Three trials have compared multiple fatigue treatments within the same population [16, 47, 48]. Krupp et al. [16] found amantadine superior to both placebo and pemoline in 93 PwMS. Ledinek et al. [48] observed amantadine to be superior to placebo in 69 PwMS after 1 month, while modafinil and acetyl‐L‐carnitine showed no significant benefit. Together, these two studies suggest a possible advantage of amantadine, yet this could not be replicated by Nourbakhsh and colleagues [47]. They conducted the most rigorous and well‐controlled study to date using a crossover design with four treatment sequences to minimize unmasking from off‐target effects and increase sample size [47]. Main findings include a clinically relevant, but similarly large reduction in self‐reported fatigue across modafinil, amantadine, methylphenidate, and placebo, suggesting that effects largely can be attributed to a psychological rather than a true pharmacological effect. Differences in blinding and study design likely contributed to inconsistencies across trials [47], and parallel‐group trials, in particular, are at higher risk of unmasking due to the noticeable side effects of these medications, which may result in false attribution of efficacy of the active drug [47, 49]. Our findings are consistent with Nourbakhsh et al., as we observed no significant differences in work loss across treatments. Moreover, approximately half of new amantadine users did not collect a second prescription within 2 years, compared with 28% of modafinil users and 15% of ADHD drug users, suggesting limited satisfaction with amantadine.

Notably, the greater attenuation of monthly work loss after treatment initiation in our study may, at least to some degree, be due to a placebo effect, as has been the case with self‐reported outcomes [47]. Even if the effect is caused by placebo, this may still support a positive effect of offering patients some symptomatic fatigue treatment, depending on what alternative forms of support are available. Moreover, the similar work loss trajectories across treatment cohorts may reflect factors other than true similarity in treatment effects. In our main analyses, we used an ever‐treated approach to minimize selection bias, but this approach may have diluted potential differences between treatments. For instance, many new amantadine users discontinued or switched to an ADHD drug after initiation, possibly inflating an effect of amantadine, as discontinuation often reflects a lack of effect.

Overall, our findings align with updated National Institute for Health and Care Excellence (NICE) guidelines, also referenced in Swedish guidelines on the management of MS fatigue [8, 9]. NICE does not recommend one fatigue treatment over another but acknowledges that pharmacological options may benefit some individuals and can be considered before non‐pharmacological approaches when rapid relief is needed [8].

## 5 Strengths and Limitations

By linking national registers to the SMSreg, we included nearly all PwMS in Sweden initiating any fatigue treatment, limiting selection bias and improving generalizability. The register linkage also allowed us to adjust for many important confounders, such as comorbidity, though residual confounding remains a limitation. Specifically, we could not adjust for fatigue severity due to a high proportion of missing data, particularly in the untreated cohort. Additionally, we lacked information on lifestyle factors such as smoking and physical activity. While these are unlikely to have strongly influenced treatment choice, they may have affected the decision to use fatigue treatment at all. To mitigate unmeasured confounding, we included several general health markers and demographic variables as proxies. Lastly, since the first 2 weeks of sickness benefit are paid by the employer and not captured in MiDAS, total work loss estimates will be underestimated.

## 6 Conclusions

Modafinil has been the most used pharmacological treatment for MS fatigue in Sweden, with a more recent increase in ADHD drug prescriptions. The observed attenuation of worsening work loss after modafinil initiation compared with matched untreated PwMS, corresponding to approximately 2 fewer days of work loss per year, was statistically significant but indicates only a minor potential clinical benefit of symptomatic drug treatment for MS fatigue. No significant differences were observed across treatments, but users of amantadine were less likely to fill a new prescription. Further studies are needed to assess the long‐term benefit–risk balance of these treatments, ideally including adverse effects (especially on the cardiovascular system), objective measures of activity level, and self‐reported fatigue scores.

## Author Contributions

S.E., J.R., T.F., and F.P. contributed to the conception and design of the study; S.E., and T.F. contributed to the acquisition and analysis; S.E., J.R., T.F., and F.P. contributed to drafting the text or preparing the figures.

## Conflicts of Interest

F.P. has received research grants from Janssen, Merck KGaA and UCB, and fees for serving on the DMC in clinical trials with Lundbeck and Roche, and preparation of an expert witness statement for Novartis; none of these activities relate to the submitted work. J.R. reported participation in research projects funded by pharmaceutical companies, all with funds paid to the institution where he is employed outside the submitted work. S.E., T.F.: declare no conflicts of interest.

## Supporting information


**Data S1:** acn370259‐sup‐0001‐DataS1.docx.

## Data Availability

Sharing of de‐identified data will be considered upon reasonable request and in accordance with current legislation regarding the protection of personal data.
