# Supplementary material for: Use of Symptomatic Drug Treatment for Fatigue in Multiple Sclerosis and Patterns of Work Loss
Source: Ann Clin Transl Neurol. 2025 Nov 20;13(4):736–46. doi: 10.1002/acn3.70259 (PMC13071106; doi:10.1002/acn3.70259)
Supplement: Supplementary file 1 — Data S1: acn370259‐sup‐0001‐DataS1.docx. [file ACN3-13-736-s001.docx]

Supplementary Material

Use of symptomatic drug treatment for fatigue in multiple sclerosis and patterns of work loss: a register-based Swedish cohort study

Contents

[**Table S1:** Detailed definitions of covariates. 2](#_Toc212198772)

[**Table S2:** Missing data table 5](#_Toc212198773)

[**Figure S1:** Standardized mean differences to the pooled population, stratified by cohort, before (upper panel) and after weighting (lower panel). 6](#_Toc212198774)

[**Figure S2:** Sensitivity analysis 1. Standardized mean differences to the pooled population, stratified by cohort before (upper panel) and after weighting (lower panel). 7](#_Toc212198775)

[**Table S3:** Results from sensitivity analysis 1. 8](#_Toc212198776)

[**Table S4:** Results from sensitivity analysis 2. 9](#_Toc212198777)

[**Figure S3:** Weighted mean monthly net days of work loss from 12 months before to 24 months after the index date, stratified by pre‑2020 (upper panel) and 2020–2023 (lower panel). 10](#_Toc212198778)

| **Table S1:** Detailed definitions of covariates. | | |
| --- | --- | --- |
| **Covariate** | **Description** | **Data source** |
| **Index year** | Continuous | The Prescribed Drug Register |
| **Socio-demographic** |  |  |
| Age at index date | Continuous | Total Population Register |
| Sex, female | Categorized into: female, male. | Total Population Register |
| Swedish-born | Y/N | Total Population Register |
| Region of residence | Categorized into: Northern Sweden, Stockholm, South-eastern Sweden, Southern Sweden, Middle Sweden, Western Sweden | Total Population Register |
| Education | Highest education achieved as recorded in the year prior to index date. Categorized into: ≤12 years or >12 years of education. | LISA |
| Unemployed | Y/N | LISA |
| Days with work loss last year | The number of net days with sick leave or disability pension | MiDAS |
| **Comorbidities** |  |  |
| Days hospitalized last 5 years | Any inpatient visits | Inpatient component of the Patient register |
| Depression | Record of depression diagnosis within 5 years prior to index date (ICD-10: F32-F34, F38-F39). Indicator variable (Y/N). | Main or contributory diagnosis in the  in- or outpatient components of the Patient register |
| Anxiety | Record of anxiety disorder within 5 years prior to index date (ICD-10: F40-F45, F48). Indicator variable (Y/N). | Main or contributory diagnosis in the  in- or outpatient components of the Patient register |
| Other psychiatric comorbidities | Record of any other psychiatric comorbidity within 5 years prior to index date (ICD-10: F00-F99 except F32-34, F38-39 and F40-48). Indicator variable (Y/N). | Main or contributory diagnosis in the  in- or outpatient components of the Patient register |
| Sleep disorders | Record of sleep disorders (except narcolepsy with or without cataplexy) within 5 years prior to the index date (ICD-10: G47.0- G47.3 and G47.8-G47.9). Indicator variable (Y/N). | Main or contributory diagnosis in the  in- or outpatient components of the Patient register |
| MACE | Record of MACE within 5 years prior to the index date (ICD-10: I20.0, I21, and I60-I64). Indicator variable (Y/N). | Main or contributory diagnosis in the  in- or outpatient components of the Patient register |
| Arrhythmia | Record of arrhythmia diagnosis within 5 years prior to the index date (ICD-10: I44, I45, I47-I49). Indicator variable (Y/N). | Main or contributory diagnosis in the  in- or outpatient components of the Patient register |
| Invasive cancer | Any invasive cancer within 5 years prior to the index date | The Cancer Registry |
| Hospitalized infection | Record of hospitalized infection within 5 years prior to the index date (ICD-10: A00-B99, D73.3, E06.0, E32.1, G00-G02, G04.2, G05-G07, H00.0, H44.0, H60.0-H60.3, H66-H67, H70, I30.1, I40.0, J00-J22, J32, J34.0, J36, J38.3, J39.0-J39.1, J44.0, J85, J86, K04.4, K04.6, K04.7, K10.2, K11.3, K12.2, K14.0, K57.0, K57.2, K57.4, K57.8, K61, K63.0, K65.0, K65.1, K65.2, K65.9, L00-L08, L30.3, M00-M01, M46.2-M46.5, M60.0, M65.0, M71.0, M71.1, M72.6, M86, N10, N11, N12, N13.6, N15.1, N15.9, N30.0 N30.8, N30.9, N34.0, N34.1, N34.2, N390, N41.2, N43.1, N45.2, N45.3, N45.4, N48.2, N61, N70, N73, N75.1). Indicator variable (Y/N). | Main diagnosis in the inpatient component of the Patient register |
| **Treatment dispensations** |  |  |
| Antidepressant use | Record of filled prescription for antidepressants within 1 year prior to the index date (ATC codes: N06AB, N06AF, N06AG, N06AX, excluding N06AX21 and N06AA, as these are commonly used to treat neuropathic pain in people with MS). Indicator variable (Y/N). | The Prescribed Drug Register |
| Anxiolytics treatment use | Record of filled prescription for benzodiazepines within 1 year prior to the index date (ATC codes: N05BA04, N05BA06, N05BA12. N05BA01 not included as commonly used to treat other symptoms in people with MS). Indicator variable (Y/N). | The Prescribed Drug Register |
| Sleeping aids treatment use | Record of filled prescription for hypnotic drugs within 1 year prior to the index date (ATC codes: N05CF01, N05CF02, N05CF03, N05CH01, N05CM06, R06AD01, R06AD02, R06AD52.) Indicator variable (Y/N). | The Prescribed Drug Register |
| Pain treatment use | Record of filled prescription for pain treatment within 1 year prior to the index date (ATC codes: M03BB, M03BC, N02A, N02BA, N02BE, N02BF01, N02BF02, N02BG10, N02C, N06AA09, N06AA10  Indicator variable (Y/N). | The Prescribed Drug Register |
| Antidiabetic use | Record of filled prescription for antidiabetics within 1 year prior to the index date (ATC code: A10). Indicator variable (Y/N). | The Prescribed Drug Register |
| Fatigue treatment order | Categorized into: 1, 2, 3 | The Prescribed Drug Register |
| **MS disease-related** |  |  |
| MS type | PPMS, RRMS or SPMS | The Swedish MS Register |
| Years since MS diagnosis | Number of years from MS diagnosis to index date. Continuous. | The Swedish MS Register |
| Any relapse last year | Any relapse within one year prior to index date. Indicator variable (Y/N). | The Swedish MS Register |
| DMT | DMT treatment at index date including: dimethyl fumarate, fingolimod, glatiramer acetate, HSCT, interferons (interferon beta-1a, peginterferon beta-1a, and interferon beta-1b), natalizumab, rituximab, teriflunomide, other and no DMT. | The Swedish MS Register |
| **Sensitivity analyses** |  |  |
| EDSS | Physical disability assessed via EDSS recorded within 180 days before index date and 15 days after index date will be considered. EDSS obtained within 90 days after a relapse are ignored. Continuous variable. | The Swedish MS Register |
| SDMT | Processing speed assessed via SDMT recorded within 180 days before index date and 15 days after index date will be considered. Continuous variable. | The Swedish MS Register |
| MSIS-29 physical | Physical impact of MS assessed via MSIS-29 recorded within 180 days before index date and 15 days after index date will be considered. Continuous variable. | The Swedish MS Register |
| MSIS-29 psychological | Psychological impact of MS assessed via MSIS-29 physical recorded within 180 days before index date and 15 days after index date will be considered. Continuous variable. | The Swedish MS Register |
| ATC = anatomical therapeutic chemical, DMT = disease modifying therapy, EDSS = Expanded Disability Status Scale, HSCT= haematopoietic stem cell transplantation, ICD, international classification of disease, LISA = the longitudinal integrated database for health Insurance and labour market studies, MACE = major adverse cardiovascular event, MiDAS = Micro Data for Analysis of the Social Insurance, MS = multiple sclerosis, MSIS-29 = MS Impact Scale, PPMS = Primary Progressive MS, RRMS = Relapsing-Remitting MS, SDMT = Symbol Digit Modalities Test, SPMS=Secondary Progressive MS. | | |

| **Table S2:** Missing data table. Missing data in absolute number and percent of observations in each cohort. | | | | |
| --- | --- | --- | --- | --- |
|  | **Cohorts** | | | |
|  | **Untreated** | **Modafinil** | **Amantadine** | **ADHD drugs^a^** |
| No. of participants | 9762 | 2162 | 462 | 424 |
| Region of residence | 3 (0.0) | 1 (0.0) | 2 (0.4) | 0 (0.0) |
| Education >12 years | 39 (0.4) | 6 (0.3) | 2 (0.4) | 0 (0.0) |
| Unemployed | 3 (0.0) | 1 (0.0) | 2 (0.4) | 7 (0.0) |
| MS type | 1036 (10.6) | 87 (4.0) | 19 (4.1) | 9 (2.1) |
| Any relapse last year | 2192 (22.5) | 342 (15.8) | 75 (16.2) | 23 (5.4) |
| EDSS | 6580 (67.4) | 1142 (52.8) | 249 (53.9) | 221 (52.1) |
| SDMT | 7374 (75.5) | 1478 (68.4) | 320 (69.3) | 246 (58.0) |
| MSIS-29 physical | 7523 (77.1) | 1494 (69.1) | 331 (71.6) | 238 (56.1) |
| MSIS-29 psychological | 7523 (77.1) | 1494 (69.1) | 331 (71.6) | 280 (56.1) |
| DMT | 2192 (22.5) | 342 (15.8) | 75 (16.2) | 23 (5.4) |
| Only variables with any missing data are shown. ADHD = attention deficit hyperactivity disorder , DMT = disease modifying therapy, EDSS = Expanded Disability Status Scale, MS = multiple sclerosis, MSIS- 29 = MS Impact Scale, SDMT = Symbol Digit Modalities Test.  ^a^Central stimulants for ADHD amfetamine, dexamfetamine, methylphenidate, and lisdexamfetamine. | | | | |

| 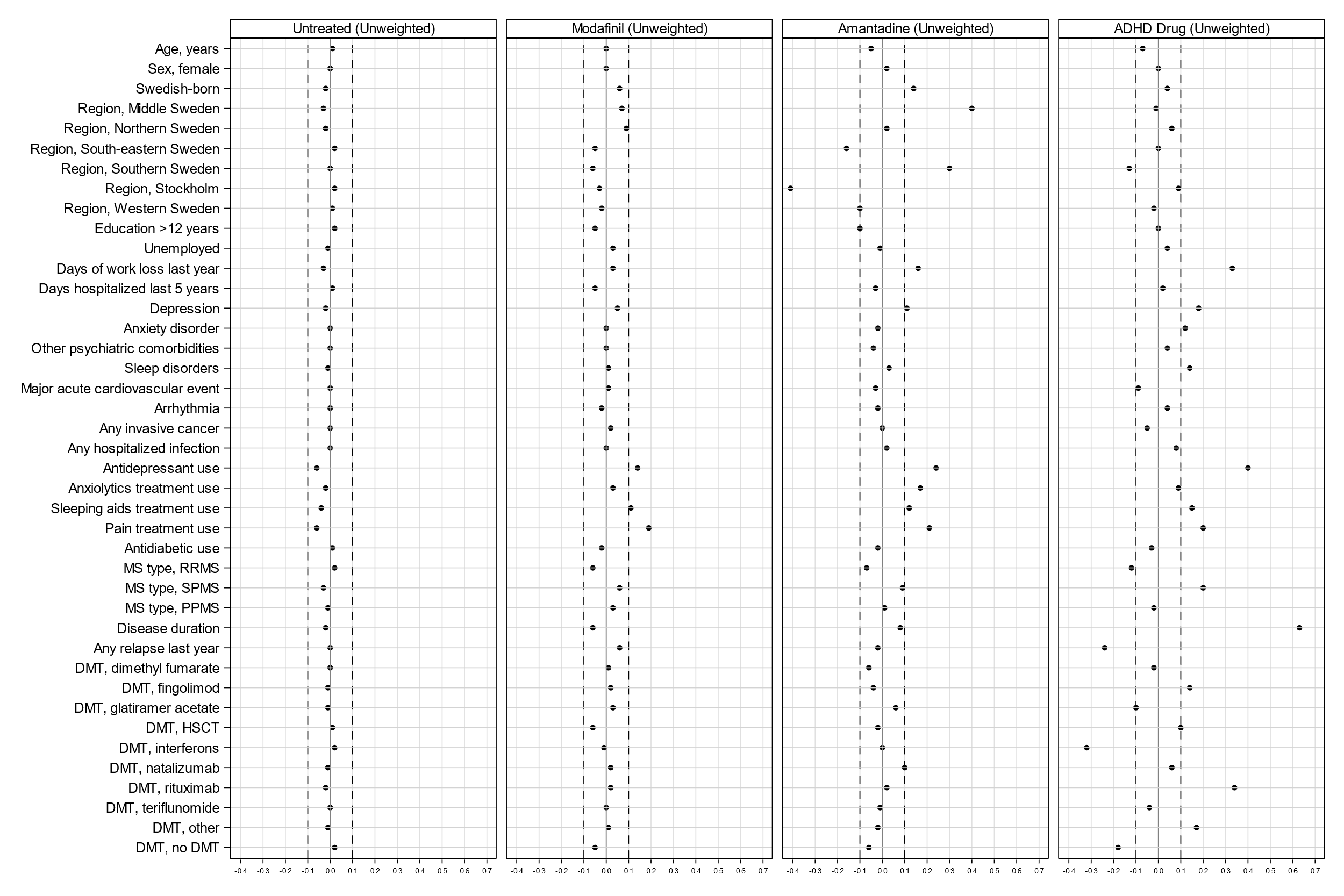 |
| --- |
| 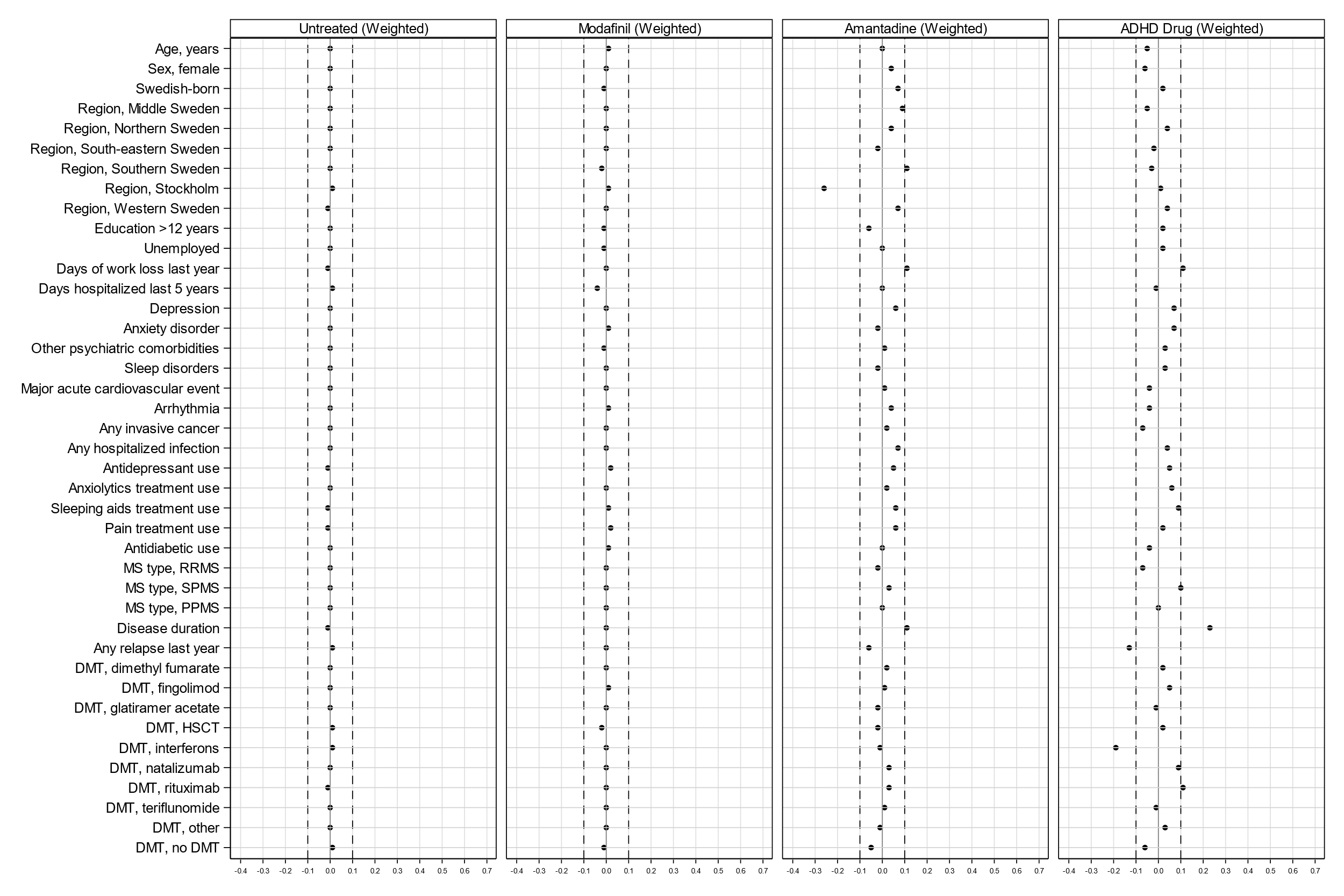 |

**Figure S1:** Standardized mean differences to the pooled population, stratified by cohort, before (upper panel) and after weighting (lower panel). Weighting was applied for age, sex, country of birth, region of residence, education, employment status, work loss, hospitalized days, depression, anxiety, other psychiatric comorbidities, sleep disorders, MACE, arrhythmia, invasive cancer, hospitalized infection, antidepressants, anxiolytics, sleeping aids, pain treatments, antidiabetics, DMT, MS type, years since MS diagnosis, any relapse last year. ADHD=attention deficit hyperactivity disorder, DMT=disease modifying therapy, MACE=major adverse cardiovascular event, MS=multiple sclerosis, PPMS=Primary Progressive MS, RRMS=relapsing-remitting MS, SPMS=secondary progressive MS.

| 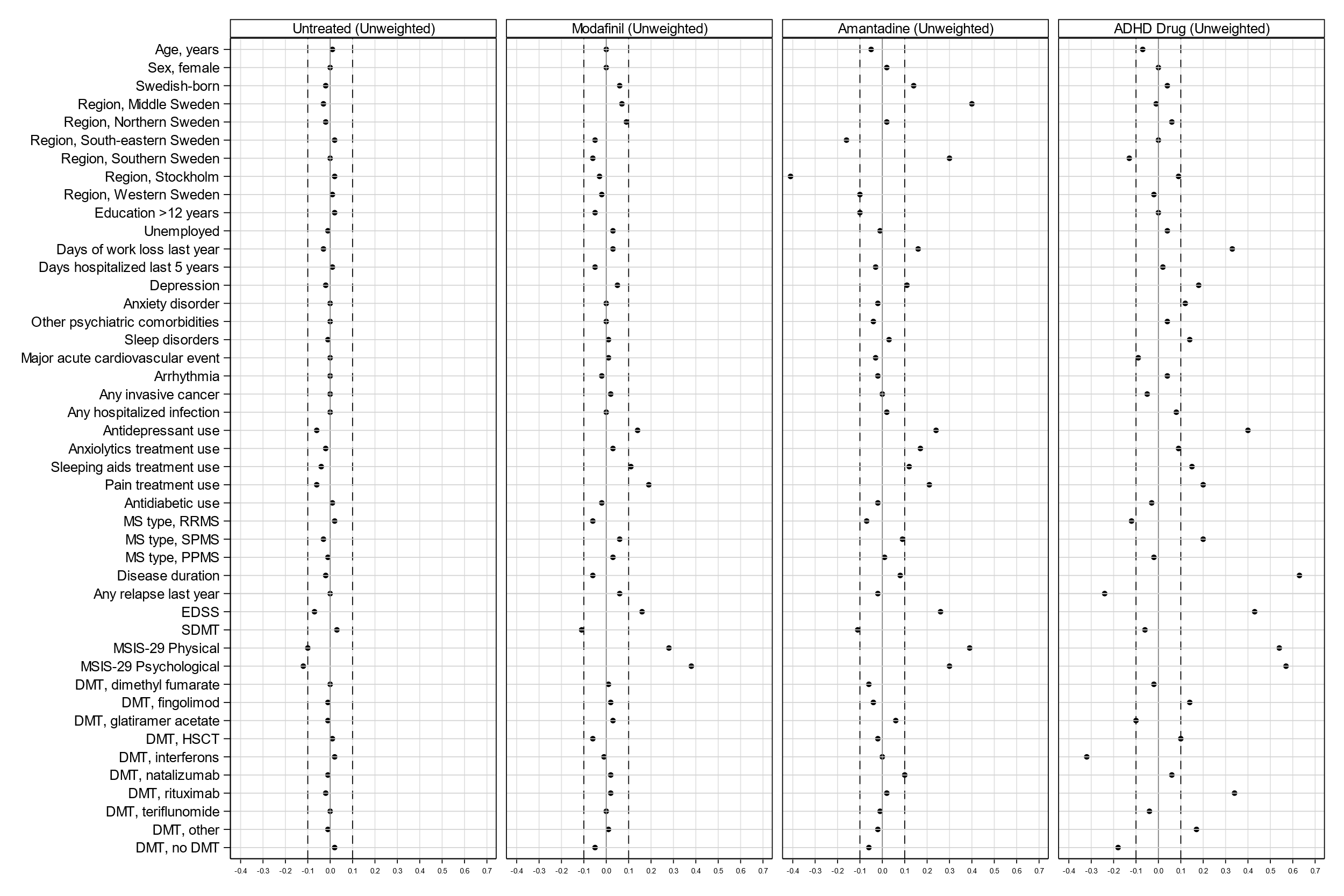 |
| --- |
| 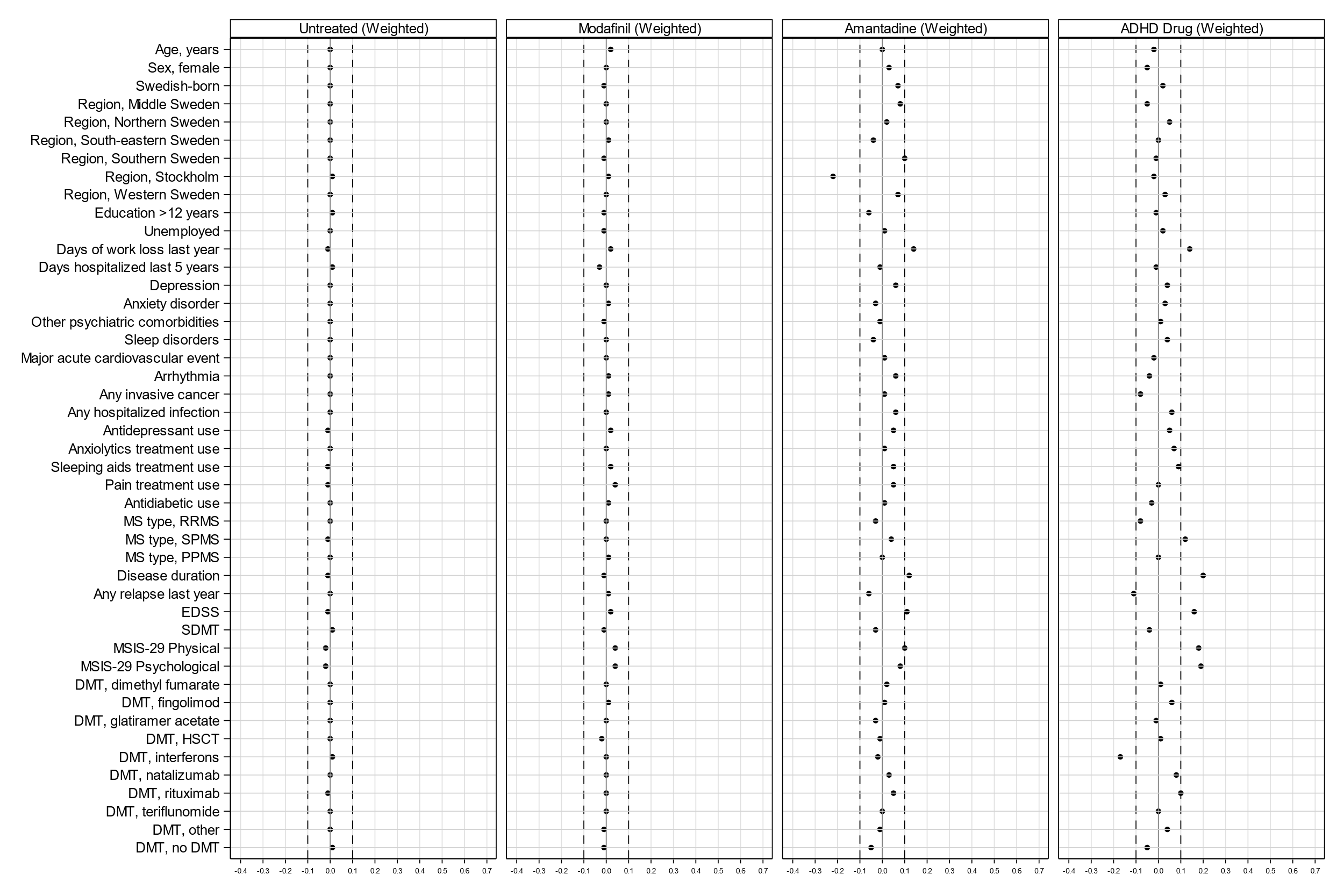 |

**Figure S2:** Sensitivity analysis 1. Standardized mean differences to the pooled population, stratified by cohort before (upper panel) and after weighting (lower panel). Weighting was applied for age, sex, country of birth, region of residence, education, employment status, work loss, hospitalized days, depression, anxiety, other psychiatric comorbidities, sleep disorders, MACE, arrhythmia, invasive cancer, hospitalized infection, antidepressants, anxiolytics, sleeping aids, pain treatments, antidiabetics, DMT, MS type, years since MS diagnosis, any relapse last year, EDSS, SDMT, MSIS-29. ADHD=attention deficit hyperactivity disorder, DMT=disease modifying therapy, EDSS=Expanded Disability Status Scale, MACE=major adverse cardiovascular event, MS=multiple sclerosis, MSIS-29=MS Impact Scale, PPMS=Primary Progressive MS, RRMS=Relapsing-Remitting MS, SDMT=Symbol Digit Modalities Test, SPMS=Secondary Progressive MS.

| **Table S3:** Results from sensitivity analysis 1. Estimates of the weighted mean monthly work loss before index, the change in work loss rates during the 12 months pre-index, and the change in work loss rates from pre-index to the 24 months post-index period, for the fatigue treatment cohorts and the untreated modafinil-matched MS cohort. The lower part of the table shows cohort differences in mean monthly work loss before index and changes in work loss rates. A positive number corresponds to a net loss of work capacity.   \|  \| **Mean Monthly Work Loss**  **(95% CI)** \| **Change in Monthly Work Loss Rates,**  **Mean (95% CI)** \| \| \| --- \| --- \| --- \| --- \| \|  \| Pre-index \| Pre-index \| Pre- to post-index \| \| **Cohort** \|  \|  \|  \| \| Untreated \| 9.97 (9.71; 10.23) \| 0.10 (0.08; 0.12) \| -0.13 (-0.15; -0.10) \| \| Modafinil \| 11.24 (10.63; 11.84) \| 0.24 (0.19; 0.28) \| -0.23 (-0.29; -0.18) \| \| Amantadine \| 12.59 (11.08; 14.10) \| 0.22 (0.11; 0.34) \| -0.21 (-0.35; -0.07) \| \| ADHD drugs^a^ \| 11.99 (10.32; 13.65) \| 0.14 (0.02; 0.26) \| -0.14 (-0.29; 0.01) \| \|  \|  \|  \|  \| \| **Comparison between cohorts** \|  \|  \|  \| \| Modafinil vs. Untreated \| 1.27 (0.61; 1.93) \| 0.14 (0.09; 0.19) \| -0.11 (-0.16; -0.05) \| \| Amantadine vs. Untreated \| 2.62 (1.09; 4.15) \| 0.13 (0.01; 0.24) \| -0.08 (-0.23; 0.06) \| \| ADHD drugs^a^ vs. Untreated \| 2.02 (0.33; 3.70) \| 0.05 (-0.07; 0.17) \| -0.01 (-0.16; 0.14) \| \| Amantadine vs. Modafinil \| 1.35 (-0.27; 2.97) \| -0.01 (-0.13; 0.11) \| 0.02 (-0.13; 0.17) \| \| ADHD drugs^a^ vs. Modafinil \| 0.75 (-1.03; 2.53) \| -0.09 (-0.22; 0.03) \| 0.09 (-0.07; 0.25) \|   Estimates are derived from a generalized estimating equations model, incorporating weights from stabilized inverse probability of treatment weighting (IPTW) to account for imbalances between cohorts at index date. Weighting was applied for age, sex, country of birth, region of residence, education, employment status, work loss, hospitalized days, depression, anxiety, other psychiatric comorbidities, sleep disorders, MACE, arrhythmia, invasive cancer, hospitalized infection, antidepressants, anxiolytics, sleeping aids, pain treatments, antidiabetics, DMT, MS type, years since MS diagnosis, any relapse last year. As a sensitivity analysis, variables with a high proportion of missing data (Expanded Disability Status Scale, Symbol Digit Modalities Test, and MS Impact Scale) were included in the IPTW model. The index date corresponds to the first filled prescription for each treatment cohort, and for the untreated cohort, it is the date of the matched modafinil prescription.  ADHD = attention deficit hyperactivity disorder, CI = confidence interval  ^a^Central stimulants for ADHD amfetamine, dexamfetamine, methylphenidate, and lisdexamfetamine. |
| --- | --- | --- | --- | --- | --- | --- | --- | --- | --- | --- | --- | --- | --- | --- | --- | --- | --- | --- | --- | --- | --- | --- | --- | --- | --- | --- | --- | --- | --- | --- | --- | --- | --- | --- | --- | --- | --- | --- | --- | --- | --- | --- | --- | --- | --- | --- | --- | --- | --- | --- | --- | --- | --- | --- | --- | --- |

| **Table S4:** Results from sensitivity analysis 2. Estimates of the weighted mean monthly work loss before index, the change in work loss rates during the 12 months pre-index, and the change in work loss rates from pre-index to the 24 months post-index period, for the fatigue treatment cohorts and the untreated modafinil-matched MS cohort. The lower part of the table shows cohort differences in mean monthly work loss before index and changes in work loss rates. A positive number corresponds to a net loss of work capacity.   \|  \| **Mean Monthly Work Loss**  **(95% CI)** \| **Change in Monthly Work Loss Rates,**  **Mean (95% CI)** \| \| \| --- \| --- \| --- \| --- \| \|  \| Pre-index \| Pre-index \| Pre- to post-index \| \| **Cohort** \|  \|  \|  \| \| Untreated \| 8.45 (6.60; 10.40) \| 0.07 (0.05; 0.09) \| -0.10 (-0.12; -0.08) \| \| Modafinil \| 9.90 (7.94; 11.87) \| 0.29 (0.24; 0.33) \| -0.27 (-0.32; -0.22) \| \| Amantadine \| 10.96 (8.63; 13.29) \| 0.26 (0.15; 0.36) \| -0.23 (-0.36; -0.10) \| \| ADHD drugs^a^ \| 10.35 (7.98; 12.72) \| 0.18 (0.08; 0.29) \| -0.18 (-0.31; -0.05) \| \|  \|  \|  \|  \| \| **Comparison**  **between cohorts** \|  \|  \|  \| \| Modafinil vs. Untreated \| 1.41 (0.80; 2.01) \| 0.21 (0.17; 0.26) \| -0.17 (-0.22; -0.12) \| \| Amantadine vs. Untreated \| 2.46 (1.07; 3.86) \| 0.19 (0.08; 0.29) \| -0.12 (-0.26; 0.01) \| \| ADHD drugs^a^ vs. Untreated \| 1.86 (0.39; 3.33) \| 0.11 (0.01; 0.22) \| -0.08 (-0.21; 0.06) \| \| Amantadine vs. Modafinil \| 1.06 (-0.42; 2.53) \| -0.03 (-0.14; 0.09) \| 0.04 (-0.10; 0.18) \| \| ADHD drugs^a^ vs. Modafinil \| 0.45 (-1.10; 2.00) \| -0.10 (-0.22; 0.01) \| 0.09 (-0.05; 0.24) \|   Estimates are derived from a generalized estimating equations (GEE) model, incorporating weights from stabilized inverse probability of treatment weighting to account for imbalances between cohorts at index date. Weighting was applied for age, sex, country of birth, region of residence, education, employment status, work loss, hospitalized days, depression, anxiety, other psychiatric comorbidities, sleep disorders, MACE, arrhythmia, invasive cancer, hospitalized infection, antidepressants, anxiolytics, sleeping aids, pain treatments, antidiabetics, DMT, MS type, years since MS diagnosis, any relapse last year. As a sensitivity analysis, region of residence, years since MS diagnosis, any relapse in the last year, and DMT were adjusted for in a doubly robust way by being included as a covariate in the GEE model in addition to the weighting. The index date corresponds to the first filled prescription for each treatment cohort, and for the untreated cohort, it is the date of the matched modafinil prescription.  ADHD = attention deficit hyperactivity disorder, CI = confidence interval  ^a^Central stimulants for ADHD amfetamine, dexamfetamine, methylphenidate, and lisdexamfetamine. |
| --- | --- | --- | --- | --- | --- | --- | --- | --- | --- | --- | --- | --- | --- | --- | --- | --- | --- | --- | --- | --- | --- | --- | --- | --- | --- | --- | --- | --- | --- | --- | --- | --- | --- | --- | --- | --- | --- | --- | --- | --- | --- | --- | --- | --- | --- | --- | --- | --- | --- | --- | --- | --- | --- | --- | --- | --- |

| **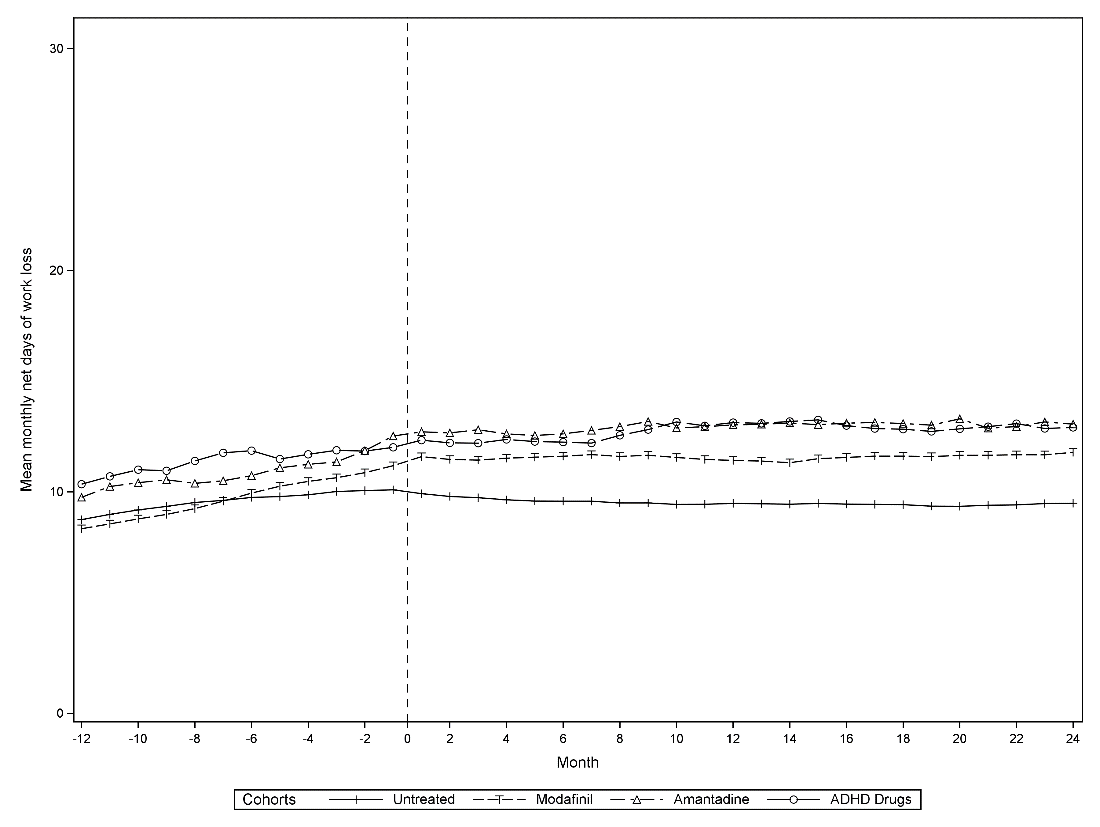** |
| --- |
| **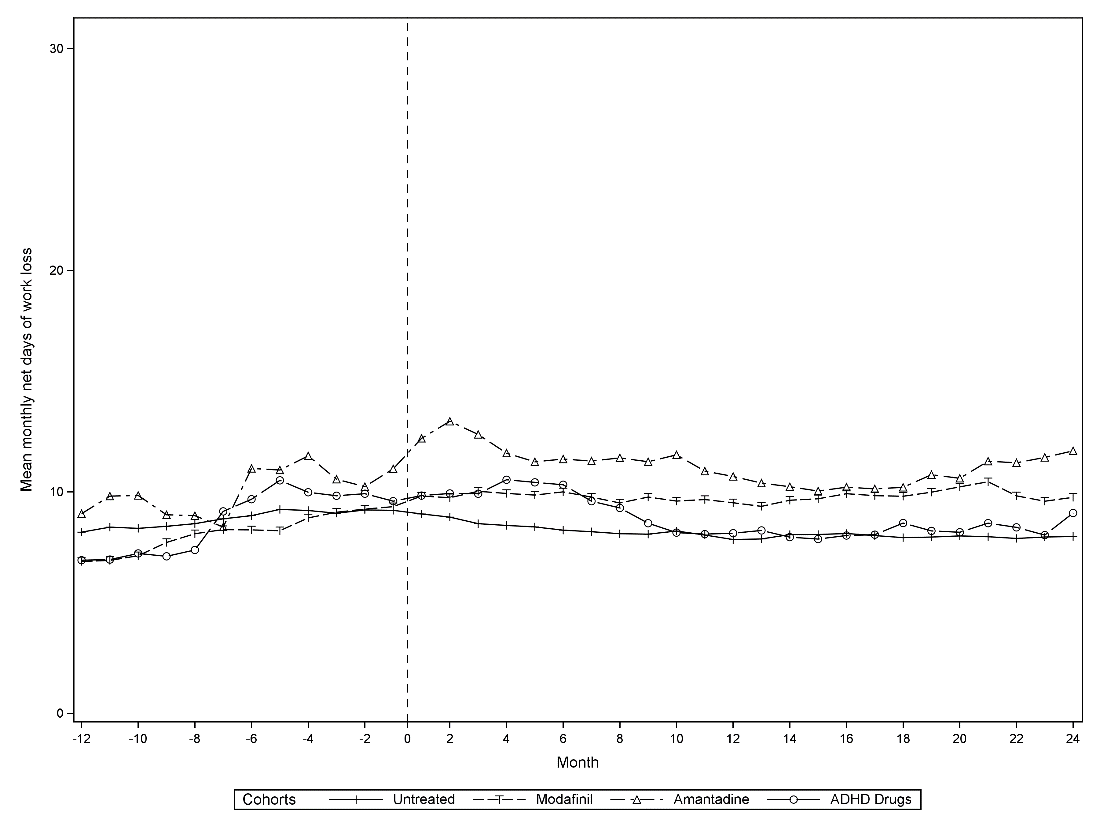** |

# **Figure S3:** Weighted mean monthly net days of work loss from 12 months before to 24 months after the index date, stratified by pre‑2020 (upper panel) and 2020–2023 (lower panel).
